# Surveillance of Multidrug-Resistant Pathogens in Neonatal Intensive Care Units of Palermo, Italy, during SARS-CoV-2 Pandemic

**DOI:** 10.3390/antibiotics12091457

**Published:** 2023-09-19

**Authors:** Giorgio Graziano, Veronica Notarbartolo, Walter Priano, Carmelo Massimo Maida, Vincenzo Insinga, Grazia Rinaudo, Arianna Russo, Roberta Palermo, Francesco Vitale, Mario Giuffrè

**Affiliations:** 1 Clinical Epidemiology Unit, University Hospital “P. Giaccone”, 90127 Palermo, Italy; giorgio.graziano@policlinico.pa.it (G.G.); carmelo.maida@unipa.it (C.M.M.); ariannarusso0111@libero.it (A.R.); francesco.vitale@unipa.it (F.V.); 2 Neonatology and Neonatal Intensive Care Unit, Ingrassia Hospital, 90132 Palermo, Italy; veronica.notarbartolo@unipa.it; 3 Department of Health Promotion, Mother and Child Care, Internal Medicine and Medical Specialties “G. D’Alessandro”, University of Palermo, 90127 Palermo, Italy; walter.priano@unipa.it (W.P.); roberta.palermo@unipa.it (R.P.); 4 Neonatology and Neonatal Intensive Care Unit, University Hospital “P. Giaccone”, 90127 Palermo, Italy; vincenzo.insinga@policlinico.pa.it; 5 Neonatology and Neonatal Intensive Care Unit, Villa Sofia Cervello Hospitals, 90146 Palermo, Italy; graziarinaudo86@gmail.com

**Keywords:** surveillance system, NICU, antimicrobial resistance, SARS-CoV-2 pandemic, epidemiological trends, bacterial carriage, multi-drug-resistant pathogens

## Abstract

Background: Antimicrobial resistance (AMR) is a topic of concern, especially in high-level care departments like neonatal intensive care units (NICUs). The systematic use of an “active” epidemiological surveillance system allows us to observe and analyze any changes in microbial distribution, limiting the risk of healthcare-associated infection (HAI) development. Methods: We have conducted a longitudinal observational study in the five NICUs of Palermo, comparing the “pre-pandemic period” (March 2014–February 2020) with the “pandemic” one (March 2020–February 2022). The primary aim of the study was to evaluate the cumulative prevalence of carriage from multi-drug resistant (MDR) bacteria in the cumulative NICUs (NICU C). Results: During the “pre-pandemic period”, 9407 swabs were collected (4707 rectal, 4700 nasal); on the contrary, during the “pandemic period”, a total of 2687 swabs were collected (1345 rectal, 1342 nasal). A statistically significant decrease in MDR-Gram-negative bacteria (GNB) carriage prevalence was detected during the pandemic. At the same time, there was a general worsening of the carriage of carbapenemase-forming MDR-GNB (CARBA-R+) and methicillin-resistant *Staphylococcus aureus* (MRSA) during the pandemic period. A significant reduction in methicillin-susceptible *Staphylococcus aureus* (MSSA) carriage was detected too. Conclusions: The surveillance of MDRO carriage in NICUs is fundamental for limiting the social and economic burden of HAIs.

## 1. Introduction

Antimicrobial resistance (AMR) is a huge global public health concern in terms of its severity increase and economic impact [1]. Thus, it involves not only high-income countries (HICs) but also low- and middle-income countries (LMICs) [2]. The spread and impact of antibiotic-resistant bacteria (ARB) in LMICs are partly explained by resource-limited hospital infrastructure, poor health system capacity, and inadequate sanitation and hygiene infrastructure [3]. In comparison to HICs, neonatal sepsis is 40 times more common in LMICs, with a mortality rate almost twice as high [4,5]. The prevalence and mortality rates vary significantly across regions, with sub-Saharan Africa, Oceania, South Asia, East Asia, and Southeast Asia carrying the highest burden. In Africa, sepsis accounts for 28% of all neonatal deaths [6]. Estimates from the European Union/European Economic Area (EU/EEA) reveal that more than 670,000 infections/year occur due to ARB, with approximately 33,000 deaths and a cost of EUR 1.1 billion [7,8].

As of 2020, in Italy, the frequencies of invasive isolates of *Klebsiella pneumoniae* resistant to third-generation cephalosporins (cefotaxime/ceftriaxone/ceftazidime) and carbapenems or other fearsome bacteria, such as methicillin-resistant *Staphylococcus aureus*, were reported as 50% and 25%, respectively [9]. Between now and 2050, from a national health system perspective, AMR will contribute to about 2500 hospital days per 100,000 inhabitants each year in our country, accounting for an annual average cost per capita of EUR 5 [10]. Both the Italian National Prevention Plan on Antimicrobial Resistance 2017–2020 (PNCAR) and the Sicilian Regional Prevention Plan 2020–2025 (PRP) include measures of control and prevention of antimicrobial resistance and healthcare-associated infections (HAIs), which comprise surveillance practices [11]. The emergence of the Coronavirus-2019 disease (COVID-19) dramatically impacted healthcare systems when the world demanded immediate, coordinated, and ambitious actions to avert the AMR crisis and the related economic and health consequences [12]. These changes resulted in modifications in routine patient care practices that could have the potential to either increase or decrease risks for HAIs [13] or patients carrying multidrug-resistant microorganisms (MDROs) [14].

Moreover, reorganizing healthcare systems for caring for patients with COVID-19 could indirectly affect the surveillance programs [15]. Among hospital patients, infants admitted to the Neonatal Intensive Care Unit (NICU), especially if preterm, could quickly develop gut microbiota dysbiosis because of an immature intestine with underdeveloped immunity, barrier function, and peristalsis [16]. Colonization with MDROs may remain unnoticed [17]. Still, in addition to other factors (i.e., gestational age, use of antimicrobials, invasive procedures, and devices), frequent contact with healthcare workers and the spread of multi-resistant infectious agents colonizing infants’ skin and mucous membranes can increase the risk of acquiring infections [18,19,20,21]. Although not all colonization leads to infection, the Gram-negative bacteria (GNB) pathogenicity may predispose preterm neonates to sepsis due to their poor immune status [22]. This study aimed to identify the epidemiological changes in MDROs’ circulation and carriage patterns considering the five NICUs of Palermo City as one intensive care unit before and during the first two years of the COVID-19 pandemic.

## 2. Results

Table 1 summarizes the absolute and relative frequencies of all isolated MDROs for cumulative NICUs (NICU C). Table 2 contains the statistical analysis results, which will be briefly illustrated below.

During the “pre-pandemic period”, 9407 swabs were collected and analyzed from all five NICUs, including 4707 rectal and 4700 nasal swabs. Among the rectal ones, we isolated 1510 MDR-GNB, 858 ESBL+ (extended-spectrum β-lactamase), and 144 CARBA R+ (carbapenemase-forming) MDR-GNB; among the nasal ones, we isolated 596 MRSA and 704 MSSA (methicillin-resistant and methicillin-susceptible *Staphylococcus aureus*). On the other hand, in the “pandemic period”, 2687 swabs were collected and analyzed, including 1345 rectal and 1342 nasal swabs. Among the rectal ones, we isolated 256 MDR-GNB isolates, 118 ESBL+, and 41 CARBA R+ MDR-GNB; among the nasal ones, we isolated 211 MRSA and 100 MSSA. 

As mentioned before, newborns in Palermo are frequently moved between the NICUs of the five city hospitals. A cumulative analysis was carried out to understand this trend, which produced the following results considering the departments as one intensive care unit. 

**Figure 1:**
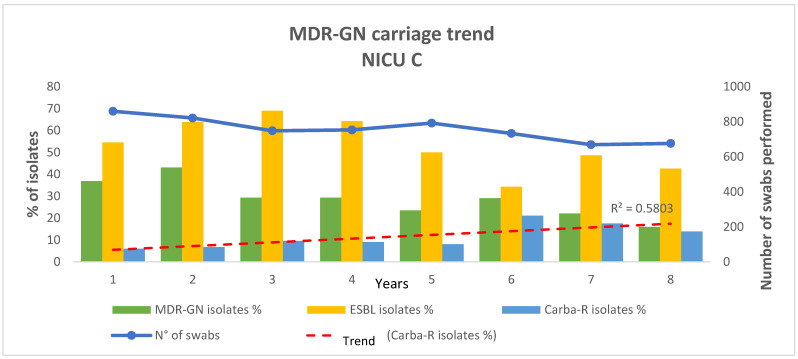
MDR-GNB carriage trend in NICU C: MDR-GN: Multi-Drug Resistant Gram Negative Bacteria; NICU: Neonatal Intensive Care Unit; ESBL: extended-spectrum β-lactamase; CARBA-R: carbapenem-resistant; N°: number. We described below the prevalence of carriage in NICU C of MDR-GNB isolates, ESBL isolates, and Carba-R isolates during the pre-pandemic and pandemic periods. The red dashed line shows the trend of Carba-R isolate carriage during the whole study period. Year 1 = March 2014–February 2015; Year 2 = March 2015–February 2016; Year 3 = March 2016–February 2017; Year 4 = March 2017–February 2018; Year 5 = March 2018–February 2019; Year 6 = March 2019–February 2020; Year 7 = March 2020–February 2021; Year 8 = March 2021–February 2022.

The carriage prevalence for MDR-GNB has decreased significantly (Figure 1). 

**Figure 2:**
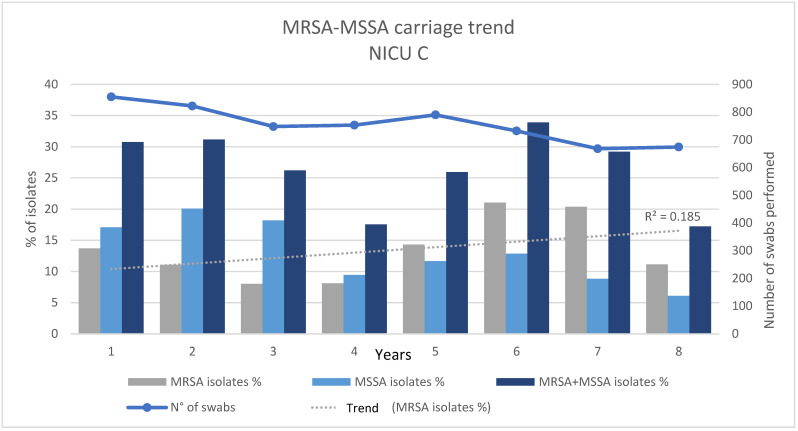
MRSA–MSSA carriage trend in NICU C: MRSA-MSSA: methicillin-resistant and methicillin-susceptible *Staphylococcus aureus*; NICU: Neonatal Intensive Care Unit; N°: number. We described below the prevalence of carriage in NICU C of MRSA isolates, MSSA isolates, and MRSA+MSSA isolates during the pre-pandemic and pandemic periods. The grey dashed line shows the MRSA isolate carriage trend during the study period. Year 1 = March 2014–February 2015; Year 2 = March 2015–February 2016; Year 3 = March 2016–February 2017; Year 4 = March 2017–February 2018; Year 5 = March 2018–February 2019; Year 6 = March 2019–February 2020; Year 7 = March 2020–February 2021; Year 8 = March 2021–February 2022.

Regarding the number of positive swabs, amelioration was observed during the pandemic period (adj-OR: 1.91 95% C.I.: 1.61–2.23; *p*-value < 0.05). When evaluating the number of isolates, an adj-OR = 2.01 (95% C.I.: 1.73–2.33; *p*-value < 0.05) was observed. Similar results were found for ESBL+ MDROs (adj-OR: 2.32, 95% C.I.: 1.89–2.0; *p*-value < 0.05 and adj-OR: 1.54, 95% C.I.: 1.18–2.01; *p*-value < 0.05, respectively). Additionally, for CARBA R+ MDR-GNB, statistical significance was reached when considering the total number of isolates (adj-OR: 0.55, 95% C.I.: 0.38–0.80; *p*-value < 0.05), with a constantly increasing trend of carriage detected during the whole study period, as illustrated in Figure 1. Finally, although with divergent odds ratios, all the variables involved for upper tract MDROs resulted significant with adj-OR *=* 0.78 (95% C.I.: 0.66–0.92; *p*-value < 0.05)*,* with a constantly increasing trend of carriage detected during the whole study period, as illustrated in Figure 2 for MRSA and with adj-OR *=* 2.20 (95% C.I.: 1.77–2.76; *p*-value < 0.05) for MSSA. All analysis results are displayed in Table 2. Epidemiological trends for upper tract MDROs are illustrated in Figure 2.

## 3. Discussion

It is well known that the emergence of antibiotic-resistant bacteria represents an urgent concern in high-intensity care settings, such as NICUs. Since the beginning of the COVID-19 pandemic, the prescription of antimicrobial drugs has increased and may have accelerated the emergence of MDR bacteria. Ineffective empiric antibiotic therapy is associated with an increased risk of morbidity and mortality, especially because it is linked to the circulation of MDROs [23], which recent studies have recognized as the major risk factor responsible for the diffusion of nosocomial infections in newborns [24]. Moreover, despite a consistent lack of scientific literature on the topic, it is well ascertained that the COVID-19 pandemic has provoked a deep change in MDROs’ circulation within NICUs during this period [25]. We detected a statistically significant decrease in the prevalence of MDR-GNB carriage during the pandemic. This reduction is not well understood to date; however, it is probably due to multiple factors: infection prevention and control strategies during the COVID-19 pandemic, antimicrobial stewardship, and national lockdowns. In fact, international and local travel are major risk factors for the transmission of MDR-GNB (i.e., ESBL-producing *Enterobacteriaceae*) [26,27,28].

Since the role of MDR-GNB intestinal carriage as a risk factor for infection has been reported in several studies [29,30,31], further studies should be performed to better understand the variable pattern mentioned above, including the settings of the different departments and clinical data of patients hospitalized during the study period. A retrospective cohort study conducted in a level 3 neonatal unit showed a drastic reduction in sepsis due to multidrug-resistant, extensively drug-resistant (XDR), and pan-drug-resistant (PDR) organisms during the COVID-19 pandemic, with a reduction in colistin use [32]. Nonetheless, in our study, NICU C displayed a general worsening of the carriage of CARBA-R+ MDR-GNB during the pandemic period, while one of the five NICUs—the only one converted to a specific COVID-19-positive patient assistive setting (COVID-19 NICU)—portrayed opposite results. The use of personal protective equipment (PPE) and stronger adherence to hygiene and infection control procedures was observed during the pandemic period; this improved the circulation of CARBA-R+ MDR-GNB in this specific setting [33]. Nevertheless, the general worsening in the prevalence of carriage of CARBA-R+ MDR-GNB in the other settings could be attributed to an increase in the use of immunosuppressive therapy, invasive devices such as ventilation, and central and peripheral catheters in non-specific COVID-19 care contexts [26]. Thus, local epidemiology, logistics, and hospital organization differences must be considered, especially during the pandemic [34]. 

To a certain extent, albeit with differences in the diverse waves of the COVID-19 pandemic, a retrospective and prospective observational epidemiological study conducted by Shbaklo et al. showing a reduction in MDRO infection during the first wave of the COVID-19 pandemic could explain the results obtained in terms of carriage reduction in our COVID-19 NICU. On the other hand, as hypothesized in a review conducted by Thoma et al. [25], the inadequate adherence to PPE, high-level use of broad-spectrum antibiotics such as carbapenem, overcrowding and minimum safety distance between patient beds, and the lack of alcohol-based sanitizer use on gloved hands, which increased the cross-contamination and cross-transmission of MDROs, could explain the NICU C results for the carriage of CARBA-R+ MDR-GNB [33]. To foster our main hypothesis that the pandemic period profoundly affected MDROs’ resistance spectrum, Witt et al. [35] showed a statistical association between COVID-19 diagnosis and drug-resistant bacteria, such as an increase in resistance to third-generation antibiotics among *Klebsiella*, *Enterobacteriaceae*, and *Pseudomonas aeruginosa* bloodstream infections in 2020 compared to 2019. In a recent observational retrospective cohort study conducted in a pediatric quaternary referral hospital in Brazil, carbapenem-resistant *Klebsiella pneumoniae* (KPC) infections associated with COVID-19 were predominant [24]. Furthermore, it was highlighted that carbapenem-resistant *Acinetobacter baumannii* (CRAB) was one of the most common pathogens associated with MDRO outbreaks during the COVID-19 pandemic in NICUs—as they can survive and persist for a prolonged time on surfaces—as seen in the results found for CARBA-R MDR-GNB carriage in the NICU C of our study [25]. The carriage of ESBL-producing MDR-GNB—a risk factor for developing ESBL infections in pediatric cardiac surgery patients [36]—diminished in NICU C during the pandemic period. Despite data limitations, another pathogen associated with MDRO outbreaks during the COVID-19 pandemic in NICUs was *Staphylococcus aureus* [24,37]. As for upper tract MDROs, our study showed a significant reduction in MSSA carriage. Contrarily, MRSA carriage significantly increased in NICU C, as a study performed by Meschiari et al. showed a significant increase in the rate of MRSA, probably due to a clonal spread during the SARS-CoV-2 pandemic [38]; the same results were registered by Ceparano et al. [39]. The rise in the carriage of MRSA during the pandemic period was probably due both to the high rate of empirical antibiotic use among infected patients, and to the reduction in active surveillance rates, isolation of MRSA carriers, and contact precautions by healthcare providers [26,40].

Certainly, some hypotheses could be made to argue about the results obtained. Surely, the implementation of face masks, the reduced number of visitations, and the heightened awareness of infection prevention practices by medical staff [41] could effectively explain the reduction in MSSA carriage. The worsening of MRSA carriage in the metropolitan area of Palermo needs to be appropriately studied and addressed. Since the COVID-19 NICU in our study experienced a reduction in all respiratory carriage indicators, a multidisciplinary quality improvement initiative aimed at infection prevention strategies could effectively lead to a significant decrease in MRSA carriage in the other settings explored. Thus, it appears reasonable to mitigate the development of antimicrobial resistance through the periodic and radicalized application of antimicrobial stewardship, infection prevention, and control policies [38]. Also, as underlined by a previous work [34], it appears fundamental to implement a coordinated strategy of control measures to reduce MDR-GNB carriage in NICU patients, with a consequent long-lasting reduction in the prevalence of MDR-GNB in an endemic setting. In fact, during the pandemic period, most MDR-GNB outbreaks occurred in NICUs [35]. Since the manipulation of patients by nurses, caregivers, and doctors, along with the environment, could be a vehicle for MDR-GNB carriage, it is fundamental to create a strong prevention policy. It could be important to use the recent World Health Organization (WHO) AWaRe (Access, Watch, Reserve) antibiotic book [42] to develop guidance for empiric antibiotic use, discontinuing antibiotic therapy after 48–72 h in the presence of infant clinical stability and negative cultural tests. Using adequate PPE, such as gloves, medical gowns, and caps, before caregiving infants, and establishing a strict flow-chart for hand-washing both have a pivotal role. Having a contact person for the correct allocation of devices and their management, establishing the correct nurse–infant ratio to improve medical assistance, and having a maximum number of beds in each room to avoid overcrowding are other important strategies that can be used. Finally, it is also important to improve environmental and water sanitation [43] with periodic checks and to ensure the “cohorting” and “superisolation” of outbreaks.

Implementing preventive interventions through periodic meetings is essential for sharing surveillance data, increasing the awareness and relevance of AMR, and discussing critical points and possible solutions. Sharing protocols, information, and experiences within the NICU network of a metropolitan area could be of utmost relevance in this sense. It is well known that the most important risk factor for outbreaks was the limitation of MDRO screening surveillance during the pandemic due to economic reasons [25]. It is, therefore, mandatory to keep our surveillance system working, as several well-known risk factors for colonization or infection with MDROs—low gestational age, low birth weight, and extended hospital stay—persist in this frail and complex population [25]. 

## 4. Materials and Methods

### 4.1. Aim of the Study 

This longitudinal observational study aimed to evaluate and compare, through the analysis of active surveillance system data, the cumulative prevalence of carriage of multi-drug resistant bacteria in 5 NICU assistive settings in the city of Palermo during the “pandemic period” (March 2020–February 2022) versus the “pre-pandemic period” (March 2014–February 2020). We expected to identify epidemiological changes in MDRO circulation and carriage patterns during the two different periods of the study, considering these wards, due to frequent transfers, as one intensive care unit. In particular, we supposed a reduction in MDRO carriage during the COVID-19 pandemic due to the use of PPE, stronger adherence to hygiene, and enhanced infection control procedures. We also used these data to offer useful information to clinicians in order to contain or avoid any epidemic outbreaks in the ward. 

### 4.2. Collection of Samples and Microbiological Analysis

The detection and subsequent identification of all MDRO specimens were obtained by analyzing samples—such as nasal and rectal swabs—collected at 4-week intervals (for study purposes referred to as a “month”) from each newborn child hospitalized at the sampling time. The specimens of interest were the following: methicillin-resistant (MRSA) and methicillin-susceptible *Staphylococcus aureus* (MSSA), multi-drug resistant Gram-negative bacteria (MDR-GNB), extended-spectrum β-lactamase (ESBL+), and carbapenemase-forming MDR-GNB (CARBA-R+).

Moreover, since transfers of patients frequently occur between the operative units mentioned above, the NICUs’ data were assessed cumulatively (NICU C). The latter evaluation was made to assess NICUs’ MDRO circulations at a metropolitan level comprehensively. During the “pre-pandemic period”, a total of 9407 swabs were collected and analyzed from all five NICUs, including 4707 rectal and 4700 nasal swabs; on the other hand, in the “pandemic period”, a total of 2687 swabs were collected and analyzed, including 1345 rectal and 1342 nasal swabs. Liquid cultures (5 mL of Brain Heart Infusion, Oxoid, Thermo Scientific, Waltham, MA, USA) were used to enrich nasal samples for 24–48 h at 37 °C. In the presence of growth, evaluated by turbidity, broth subcultures were plated on mannitol salt agar (MSA). After a 48 h incubation at 37 °C, suspected MRSA colonies, identified by their golden yellow pigment with an opaque halo, were sub-cultured in Mueller–Hinton Agar, Oxoid (Thermo Scientific, Waltham, MA, USA) plates supplemented with oxacillin (6 μg/mL) and incubated overnight at 37 °C. Colonies that meet the previously mentioned criteria but test negative on the incubation test are designated MSSA. For MRSA-classified species, antibiotic sensitivity to gentamicin (10 μg), tetracycline (10 μg), and cefoxitin (30 μg) was tested using the agar diffusion method (Kirby–Bauer disk diffusion). Inhibition halos were then interpreted following the European Committee on Antimicrobial Susceptibility Testing (EUCAST) guidelines [44]. Rectal samples were enriched in liquid cultures (5 mL of Brain Heart Infusion, Oxoid, Thermo Scientific, Waltham, MA, USA) for 24 h at 37 °C, then plated in McConkey Agar filled with three antimicrobial discs (amoxicillin–clavulanate 30 μg, meropenem 10 μg, ceftazidime 30 μg) to detect multi-drug resistant bacteria [45]. After overnight incubation, suspected MDR colonies were isolated for identification (standard biochemical methods), susceptibility testing, and ESBL detection according to the European Committee on Antimicrobial Susceptibility Testing (EUCAST) guidelines [44,46,47]. MDR-GNB were defined as Gram-negative bacteria that, in vitro, did not respond to at least three different classes of antimicrobials under testing (aminopenicillins, third-generation cephalosporins, monobactams, aminoglycosides, and carbapenems) [48,49,50].

### 4.3. Statistical Analysis

The statistical analysis was carried out using the R^©^ statistical software package (version 3.6.2, accessed on 23 May 2023). For descriptive and inferential analysis, we considered the following variables: for MDR-GNB, either the number of MDR-GNB positive swabs or the number of MDR-GNB isolates on the total number of rectal swabs performed; for ESBL+ and CARBA R+ MDR-GNB, we took under consideration the number of isolates either on the total number of rectal swabs or on the total number of MDR-GNB isolates; for MRSA/MSSA, the analysis was performed considering these MDROs both singularly and cumulatively. 

Moreover, evaluations were made solely on the total number of nasal swabs performed. For rectal tract MDR-GNB, ESBL+, and CARBA R+ MDR-GNB, we considered either the absolute number of positive swabs, or, as it is possible to isolate more than one MDR-GNB specimen per single swab analyzed, the total number of isolates. On the other hand, the same evaluation was not applied to upper respiratory tract MDROs. The specimen we searched for was, in fact, *Staphylococcus aureus.* It could be possible to isolate, from a single swab, both MSSA and MRSA. In that case, the MDROs mentioned above were counted, for analysis purposes, individually and cumulatively. The chi-squared test and Fisher’s exact test were performed to assess the relationship between categorical variables. Significance was previously defined as *p*-values (*p*) < 0.05 with a 95% confidence interval (C.I.). 

Odds ratio < 1 values showed that being hospitalized during the “pre-pandemic period” was protective for MDRO carriage compared to the “pandemic period”, and vice versa, showing risk for MDRO carriage for odds ratio > 1 values. Descriptive and inferential analyses are summarized in Table 1 and Table 2, respectively. 

## 5. Conclusions

As we previously highlighted, the active surveillance program in the NICUs of the Palermo metropolitan area is helpful in providing a description and a general vision of the carriage of MDROs in individual settings and in the whole metropolitan area, thus highlighting the general worsening of CARBA-R+ MDR-GNB, and MRSA circulation during the SARS-CoV-2 pandemic period. To this end, further studies are needed to assess the quantity of carriage-to-infection transition. It also appears of utmost relevance to establish, implement, maintain, and control specific procedures, such as correct antimicrobial stewardship for drug doses, the duration and administration route of therapy, proper hand hygiene sanitation methods and procedures, appropriate crowding of the assistive settings, minimum safety distance between patient beds, use of alcohol-based sanitizer and change of gloves after every patient visit, which are useful for preventing and controlling infectious diseases by identifying new carriage clusters or changes in carriage patterns in these specific settings. 

## Figures and Tables

**Table 1:** Absolute and relative frequencies of all isolated MDROs (MDR-GNB, ESBL, CARBA-R, MRSA, and MSSA) for cumulative Neonatal Intensive Care Units (NICU C) included in the two different study periods (March 2014–February 2019 vs. March 2020–February 2022).

		NICU C	
		March 2014–February 2019 “Pre—Pandemic Period”	March 2020–February 2022 “Pandemic Period”
	**Number of rectal swabs**	4707	1345
**MDR-GNB**	Number of MDR-GNB positive swabs	1386	241
	Number of MDR-GNB isolates	1510	256
	% (MDR-GNB isolates/Number of rectal swabs)	32.08	19.03
**ESBL+**	Number of ESBL+ isolates	858	118
	% (ESBL+ isolates/Number of MDR-GNB isolates)	56.82	46.09
	% (EBSL+ isolates/Number of rectal swabs)	18.23	8.77
**CARBA-R+**	Number of CARBA-R+ isolates	144	41
	% (CARBA-R+ isolates/Number of MDR-GNB isolates)	9.54	16.02
	% (CARBA-R+ isolates/Number of rectal swabs)	3.06	3.05
	**Number of nasal swabs**	4700	1342
**MRSA/MSSA**	Number of MRSA isolates	596	211
	% (MRSA isolates/Number of nasal swabs)	12.68	15.72
	Number of MSSA isolates	704	100
	% (MSSA isolates/Number of nasal swabs)	14.98	7.45
	Number of MRSA and MSSA isolates	1300	311
	% (MRSA and MSSA isolates/Number of nasal swabs)	27.66	23.17

NICU: Neonatal Intensive Care Unit; MDR-GNB: Multi-Drug Resistant Gram Negative Bacteria; ESBL: extended-spectrum β-lactamase; CARBA-R: carbapenem-resistant; MRSA/MSSA: methicillin-resistant and methicillin-susceptible *Staphylococcus aureus*; +: positive.

**Table 2:** Statistical analysis results related to the comparison between the prevalence of carriage of MDROs (MDR-GNB, ESBL, CARBA-R, MRSA, and MSSA) for cumulative Neonatal Intensive Care Units (NICU C) included in the two different study periods (March 2014–February 2019 vs. March 2020–February 2022).

		NICU C	
		***p*-Value** March 2014–February 2019 vs. March 2020–February 2021	adjOR–95% C.I.
**MDR-GNB**	Number of MDR-GNB positive swabs/Number of rectal swabs	***p* < 0.0001**	1.91 (1.61–2.23)
	Number of MDR-GNB isolates/Number of rectal swabs	***p* < 0.0001**	2.01 (1.73–2.33)
**ESBL+**	Number of EBSL+ isolates/Number of rectal swabs	***p* < 0.0001**	2.32 (1.89–2.84)
	Number of ESBL+ isolates/Number of MDR-GNB isolates	***p* < 0.05**	1.54 (1.18–2.01)
**CARBA-R+**	Number of CARBA-R+ isolates/Number of rectal swabs	*p* = 0.96	
	Number of CARBA-R+ isolates/Number of MDR-GNB isolates	***p* < 0.05**	0.55 (0.38–0.80)
**MRSA/MSSA**	Number of MRSA isolates/Number of nasal swabs	***p* < 0.01**	0.78 (0.66–0.92)
	Number of MSSA isolates/Number of nasal swabs	***p* < 0.0001**	2.20 (1.77–2.76)
	Number of MRSA + MSSA isolates/Number of nasal swabs	***p* < 0.01**	1.27 (1.09–1.46)

NICU: Neonatal Intensive Care Unit; adjOR: adjusted odds ratio; C.I.: confidence interval; MDR-GNB: Multi-Drug Resistant Gram Negative Bacteria; ESBL: extended-spectrum β-lactamase; CARBA-R: carbapenem-resistant; MRSA/MSSA: methicillin-resistant and methicillin-susceptible *Staphylococcus aureus*; +: positive.

## Data Availability

Not applicable.
